# The Solidarity and Health Neutrality of Physicians in War & Peace

**DOI:** 10.1371/currents.dis.1a1e352febd595087cbeb83753d93a4c

**Published:** 2017-01-20

**Authors:** Frederick M. Burkle, Timothy Erickson, Johan von Schreeb, Stephanie Kayden, Anthony Redmond, Emily YY Chan, Francesco Della Corte, Hilarie Cranmer, Yasuhiro Otomo, Kirsten Johnson, Nobhojit Roy

**Affiliations:** Harvard Humanitarian Initiative, Harvard University, Cambridge, Massachusetts, USA; The Woodrow Wilson International Center for Scholars, Washington, DC, USA; ChiefBrigham and Women's Hospital, Harvard Medical School, Harvard Humanitarian Initiative, Harvard University; Centre for Research on Health Care in Disasters, Health System and Policy, Department of Public Health Sciences, Karolinska Institutet, Stockholm, Sweden; ChiefBrigham and Women's Hospital, Harvard medical School; Humanitarian and Conflict Response Institute, University of Manchester, Manchester, UK; Collaborating Centre for Oxford University and CUHK for Disaster and Medical Humanitarian Response (CCOUC), JC School of Public Health and Primary Care, The Chinese University of Hong Kong, Hong Kong S.A.R., China, Nuffield Department of Medicine, University of Oxford, Oxford, United Kingdom, FXB Centre of Health and Human Rights, Harvard University, Cambridge, Massachusetts, USA; Research Center in Emergency and Disaster Medicine and Computer Science applied to Medicine (CRIMEDIM), Università del Piemonte Orientale, Novara, Italy; Director, Global Disaster ResponseMassachusetts General Hospital; Professor and ChairmanTokyo Medical and Dental University; McGill University; Centre for Research on Health Care in DisastersKarolinska Institutet

## Abstract

The wars in the Middle East have led to unprecedented threats and attacks on patients, healthcare workers, and purposeful targeting of hospitals and medical facilities. It is crucial that every healthcare provider, both civilian and military, on either side of the conflict become aware of the unique and inherent protections afforded to them under International Humanitarian Law. However, these protections come with obligations. Whereas Governments must guarantee these protections, when violated, medical providers have equal duty and obligations under the Law to ensure that they will neither commit nor assist in these violations nor take part in any act of hostility. Healthcare providers must not allow any inhuman or degrading treatment of which they are aware and must report such actions to the appropriate authorities. Failure to do so leads to risks of moral, ethical and legal consequences as well as penalties for their actions and inactions. There must be immediate recognition by all parties of the neutrality of health care workers and their rights and responsibilities to care for any sick and injured patient, regardless of their nationality, race, religion, or political point of view.

## Commentary

As physicians, we are reminded of Sir William Osler’s 1906 speech to graduating medical students when he asserted that: “*Medicine is the only world-wide profession, following everywhere the same methods, actuated by the same ambitions, and pursuing the same end. This homogeneity, its most characteristic feature, is not shared by the law or religion”, nor the “extraordinary solidarity which makes the physician at home in any country*.”^1^

In the century that followed, no other profession has exhibited the influential number of international associations, societies and collaborative efforts as has medicine. The end of the Cold War brought multiple humanitarian crises and the development of international academic and educational training programs designed to prepare humanitarian workers for these complex global tasks. The rigorous curriculum includes the unique roles and responsibilities that healthcare providers have under the Post-World War II Geneva Convention (GC) and International Humanitarian Law (IHL) which ensures their right to international health neutrality.

Historically, war has caused the loss of countless lives and has ravaged the most vulnerable populations, especially women, children, the elderly and disabled. The humanitarian crises that wars create, and the manner in which the world responds, have changed with every generation.^2^ Present-day healthcare workers in public hospitals and non-governmental organizations in conflict zones have never faced greater risks. In fact, deaths among all humanitarian workers have outnumbered those of UN Peacekeepers in recent years.^3^

The cross-border territorial wars that dominated the 20th Century have been replaced by endless religious, ethnic and tribal conflicts that produce neither winners nor losers. The war in Afghanistan has raged on since 1979, in Somalia since 1991, and the recent Yemeni civil war since 2015. Yet, there is something especially abhorrent about the current war in Syria that should gain the attention of every healthcare provider and health policy maker. These alarming statistics are reminders of the egregiously barbaric quest of ancient wars, designed to ensure that no survivor would remain to take revenge:

*As of June 2016, 757 healthcare personnel have been killed and 382 premeditated attacks have occurred on 269 separate medical facilities across Syria. One-hundred and twenty-two hospitals have been targeted multiple times*.^4^ *In the midst of this misery one cannot forget the US targeted aerial bombing of Médecins Sans Frontières hospital in Kunduz Afghanistan in October 2015 that killed 42 people, including 14 MSF staff members, and wounded dozens more*.^5^

We must take notice and stand in solidarity with our healthcare colleagues in Syria where patients, healthcare workers, and hospitals are under constant threat and attack. We have the obligation to ensure that every health care provider, both civilian and military, on either side of the current conflict in Syria, be made aware of the inherent protections provided them under IHL, including the four GCs of 1949, as well as the principles and rules of IHL applicable to the conduct of hostilities, which include the targeting of hospitals and medical facilities. These principles and rules must be upheld.

Healthcare providers of the militaries involved on both sides of the conflict are granted provisions and protections under international laws clearly referenced in The Hague Statement on Respect for Humanitarian Principles (1991), UN Security Council Resolution 2286 on attacks against medical workers (2016) and military manuals of many States. As an example, the Russian Federation’s Military Manual (1990) states that attack against medical personnel constitute a prohibited method of warfare. The Russian Federation’s Regulations on the Application of IHL (2001) states: “Persons protected by international humanitarian law include medical and religious personnel. Attacks against such persons are prohibited.”

As such, healthcare providers, both civilian and military, including the Assad Government and the Russian Federation, must equally recognize that under IHL, their own medical military personnel, activities, units, transports, and hospitals are guaranteed protection against direct attack themselves (see rules 25 to 30 of the ICRC’s customary IHL study, as well as protections in the Geneva Conventions and their Additional Protocols). IHL requires Parties to ensure respect for IHL, including under common Article 1 to the four GCs. Failure to do so leads to risks of moral, ethical and legal consequences as well as penalties for their actions and inactions.

The World Medical Association (WMA) Regulations in Times of Armed Conflict and Other Situations of Violence^6^ assert that under IHL, healthcare providers can neither commit nor assist violations of IHL, nor take part in any act of hostility; must advocate and provide effective and impartial care to the wounded and sick (without reference to any ground of unfair discrimination, including whether they are the "enemy"); must remind their authorities of their obligation to search for the wounded and sick and to ensure access to health care without unfair discrimination; must not allow any inhuman or degrading treatment of which physicians are aware; must report to a commander or to other appropriate authorities if health care needs are not met; and must give consideration to how health care personnel might shorten or mitigate the effects of the violence in question, for example by reacting to violations of international humanitarian law or human rights law.

WMA summarizes the ethical obligations of physicians as follows:^6^

*During times of armed conflict and other situations of violence, standard ethical norms apply, not only in regard to treatment but also to all other interventions. The medical duty to treat people with humanity and respect applies to all patients. Governments, armed forces and others in positions of power should comply with the GC to ensure that physicians and other health care professionals can provide care to everyone in need in situations of armed conflict and other situations of violence. This obligation includes a requirement to protect health care personnel and facilities. Privileges and facilities afforded to physicians and other health care professionals in times of armed conflict and other situations of violence must never be used other than for health care purposes.*

*Physicians should recognize the special vulnerability of some groups, including women and children. Provision of such care should not be impeded or regarded as any kind of offence, nor must they ever prosecuted or punished for complying with any of their ethical obligations. Physicians have a duty to press governments and other authorities to ensure they are planning for the protection of the public health infrastructure and for any necessary repair in the immediate post-conflict period. Necessary assistance, including unimpeded passage and complete professional independence, must be granted.*

*Healthcare Parties must be Identified and protected by internationally recognized symbols such as the Red Cross, Red Crescent or Red Crystal. Hospitals and health care facilities situated in areas where there is either armed conflict or other situations of violence must be respected by all combatants. Physicians must be aware that, during armed conflict or other situations of violence, health care becomes increasingly susceptible to unscrupulous practice and the distribution of poor quality / counterfeit materials and medicines, and must attempt to take action on such practices. As such, the WMA supports the collection and dissemination of data related to assaults on physicians, other health care personnel and medical facilities, by an international body. Assaults against medical personnel must be investigated and those responsible must be brought to justice.*

In Syria, Yemen, and Afghanistan as well as in many other nations in conflict around the world, the following remedies must be implemented immediately:

1. The establishment of healthcare safe zones in conflict regions to ensure the integrity of hospitals, clinics, and medical centers.

2. Allowing safe and unfettered passage of medical supplies, equipment, and personnel.

3. Cessation of all attacks on patients, pre-hospital personnel, and hospital medical staff.

4. Recognition by all parties of the neutrality of health care workers and their rights and responsibilities to care for any sick and injured patient, regardless of their nationality, race, religion, or political point of view.

The authors are representatives of academic training centers worldwide that provide global health professionals with education and training in humanitarian assistance where every healthcare provider, both civilian and military, is made aware of the inherent protections accorded to them under international law.^7^ In the same Oslerian spirit of over a century past, we condemn absolutely these deplorable actions in Syria and demand their immediate cessation; and we ask healthcare providers globally to do the same.

## Competing Interests

The opinions expressed in this article are the authors's own and do not reflect the view of their affiliated institutions.

## Corresponding Author

Frederick M. Burkle, Jr.: skipmd77@aol.com
